# Origin, diversity, and maturation of human antiviral antibodies analyzed by high-throughput sequencing

**DOI:** 10.3389/fmicb.2012.00277

**Published:** 2012-08-02

**Authors:** Ponraj Prabakaran, Zhongyu Zhu, Weizao Chen, Rui Gong, Yang Feng, Emily Streaker, Dimiter S. Dimitrov

**Affiliations:** ^1^CCR Nanobiology Program, Protein Interactions Group, Frederick National Laboratory for Cancer Research, National Institutes of Health (NIH), FrederickMD, USA; ^2^Basic Research Program, Science Applications International Corporation-Frederick, Inc., NCI-Frederick, FrederickMD, USA

**Keywords:** HIV-1, vaccine, monoclonal antibody, IgM, immunogen, 454 sequencing

## Abstract

Our understanding of how antibodies are generated and function could help develop effective vaccines and antibody-based therapeutics against viruses such as HIV-1, SARS coronavirus (SARS CoV), and Hendra and Nipah viruses (henipaviruses). Although broadly neutralizing antibodies (bnAbs) against the HIV-1 were observed in patients, elicitation of such bnAbs remains a major challenge when compared to other viral targets. We previously hypothesized that HIV-1 could have evolved a strategy to evade the immune system due to absent or very weak binding of germline antibodies to the conserved epitopes that may not be sufficient to initiate and/or maintain an effective immune response. To further explore our hypothesis, we used the 454 sequence analysis of a large naïve library of human IgM antibodies which had been used for selecting antibodies against SARS CoV receptor-binding domain (RBD), and soluble G proteins (sG) of henipaviruses. We found that the human IgM repertoires from the 454 sequencing have diverse germline usages, recombination patterns, junction diversity, and a lower extent of somatic mutation. In this study, we identified antibody maturation intermediates that are related to bnAbs against the HIV-1 and other viruses as observed in normal individuals, and compared their genetic diversity and somatic mutation level along with available structural and functional data. Further computational analysis will provide framework for understanding the underlying genetic and molecular determinants related to maturation pathways of antiviral bnAbs that could be useful for applying novel approaches to the design of effective vaccine immunogens and antibody-based therapeutics.

## Introduction

Broadly neutralizing antibodies (bnAbs) against the HIV-1 are relatively rarely observed in patients; however, discovering HIV-1 vaccine candidates to elicit such bnAbs remains a challenge due to the extensive genetic sequence variability and complex immune evasion strategies of the HIV-1 (Burton, 2002; Johnson and Desrosiers, 2002; Haynes and Montefiori, 2006; Prabakaran et al., 2007). Among the different factors thwarting the induction of bnAbs, we previously found that all known HIV-1 bnAbs are highly divergent from germline antibodies; germline antibodies of bnAbs could not bind to the epitopes of respective mature antibodies, which led to a hypothesis that HIV-1 may have evolved to use the “holes” (absence of or weak binding to germline-lineaged bnAbs) in the human germline B cell receptor repertoire (Xiao et al., 2009). Consistent with our earlier hypothesis, we did not find any specific binders against the HIV-1 envelope glycoproteins (Envs) but only identified binders against the SARS CoV receptor-binding domain (RBD), and soluble Hendra virus G protein (sG) when combinatorial phage display libraries mimicking human antibody repertoire constructing from human IgM libraries had been used for panning experiments (Chen et al., 2012). These findings had indicated that the major problem could be related to a high level of somatic mutations required for bnAbs to accurately target the conserved structures on the HIV-1 Envs.

In this article, we have used high-throughput 454 sequencing of a large naïve library of human IgM antibodies to explore antibody repertoire landscape for finding germline usages, somatic mutations, intermediates, and phylogenetic relationships between the intermediates and corresponding antiviral-related bnAbs including the HIV-1, SARS CoV, and henipaviruses. This study helped to identify germline predecessors of bnAbs observed in normal individuals, and find maturation pathways of antiviral bnAbs. Indeed, most of the known HIV-1 bnAbs are highly divergent from their closest respective germlines as well as their intermediates as they undergo somatic mutations required for their neutralization function. The results corroborate that the HIV-1 may use a strategy to eliminate strong binding of germline antibodies due to the absence of closer anti-HIV antibody intermediates as an escape mechanism from adaptive immune responses, and finding of closer intermediates of bnAbs from rare individuals might help designing the effective vaccines against the HIV-1 and other viral diseases.

## Materials and methods

### PCR amplification and high-throughput 454 sequencing

To amplify IgM antibody sequences, cDNA was prepared from peripheral blood B cells of 10 healthy donors as received under the Research Donor Program of Frederick National Laboratory for Cancer Research, USA, which we previously used to construct a naïve human Fab phage display library for selecting antibodies against SARS CoV and henipaviruses. The complete set of primers used in the PCR amplification of IgM-derived heavy and light chains were described in detail elsewhere (Zhu and Dimitrov, 2009). For 454 sequencing, primer combinations used to amplify cDNA in separate reactions included the Roche A and B adaptor sequences along with target amplification sequence for heavy and light chain variable domains. The gene fragments were amplified in 20 cycles of PCR using the High Fidelity PCR Master from Roche. More detailed description of 454 sequencing can be found in our recent articles (Prabakaran et al., 2011, 2012). The standard Roche 454 GS Titanium shotgun library protocol was adapted as found in the Roche sequencing technical bulletin.

### Databases and tools

For quality control of antibody sequences, we trimmed the 454 sequence data and retained only sequences of length more than 300 nucleotides (nt), covering the entire antibody variable domains consisting of the three complementarity determining regions (CDR) along with framework regions (FR). We used IMGT/HighV-QUEST (Alamyar et al., 2012), a high-throughput version for deep sequencing NGS data analysis resource for the immunogenetic analysis. The output results from the IMGT/HighV-QUEST analysis in CSV files were stored at PostgreSQL database, and Structured Query Language (SQL) was used to retrieve the data for the further analysis. Heatmap generation and statistical calculations involving distributions of antibody HCDR3 lengths and mutations were carried out using SAS JMP10® statistical software (SAS Institute, Cary, NC).

### Computational analyses of antibody sequences

Translated heavy and light chain variable sequences from the 454 sequencing that shared the IGHV genes of selected antiviral antibodies and associated immunogenetics data including the details of germlines, HCDR3 lengths, and mutations were retrieved from the database by using SQL. Sequence identities between the 454 sequence data and germlines were calculated based on the pairwise alignment using local BLAST as implemented in BioEdit v7.0.9 (Hall, 1999). Phylogenetic analysis was carried out using the Archaeopteryx software (Han and Zmasek, 2009).

## Results

### Germline gene usages of antiviral bnAbs

**Figure 1:**
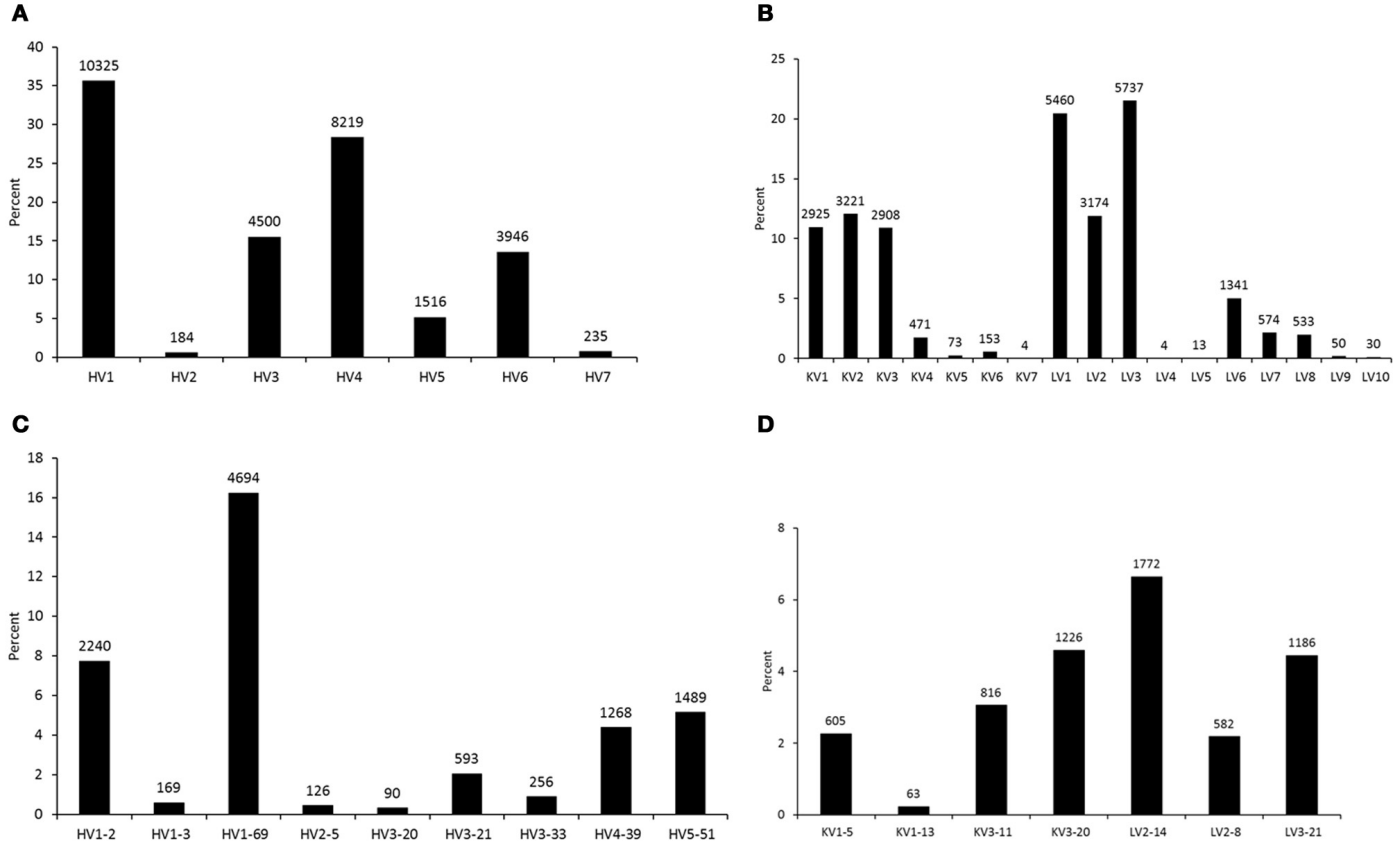
**Germline usage frequencies.** The heavy (HV) and light (KV for κ and LV for λ) chains as observed in the 454 sequencing of a human naïve IgM library are shown in **(A)** and **(B)**, respectively. The frequencies of V-genes from heavy and light chains related to the antiviral bnAbs are shown in **(C)** and **(D)**, respectively. Data labels indicate the number of unique sequences at the top of data points in bar plots.

To analyze germline origin of antiviral antibodies against the HIV-1, SARS CoV, and henipaviruses as expressed in the human IgM repertoire, we performed 454 sequencing of a non-immune library which was previously constructed from peripheral blood B cells of 10 healthy donors and used to select antibodies against SARS CoV and henipaviruses (Prabakaran et al., 2006; Zhu et al., 2006). A total of 113,139 sequences were obtained from which 91,528 sequences were found as unique with each had >300 nt in length. The total number of unique amino acid (aa) sequences for each V-gene subgroup in heavy and light chains that were found functionally productive as determined by IMGT/HighV-QUEST (Alamyar et al., 2012) are shown in Figures 1A,B, respectively. The read coverage or gene frequencies observed in the study suggested for biased germline usages and were comparable to the previous studies (Glanville et al., 2009; Prabakaran et al., 2012) but way far less than the theoretical diversity attainable by antibodies attributing to several factors such as library sampling, primer efficiency, and sequencing errors and limitations. Nevertheless, we selected known bnAbs against the viral targets including the HIV-1, SARS CoV, and henipaviruses (Table 1), and created sequence data sets related to those bnAbs from the 454 analysis as depicted in Figures 1C,D showing the germline usage frequencies of IGHV genes in the V_H_ domains, IGKV, and IGLV genes in the V_κ_ and V_λ_ domains, respectively. We found that while all antiviral-related germlines were expressed in human IgM repertoire, some preferential germline usages were noted, for example, HV1-69 gene in IGHV subgroups and KV3-20/LV2-14 genes in IGKV/IGLV subgroups were overrepresented (Figures 1C,D).

**Table 1:** **Antiviral bnAbs against the HIV-1, SARS CoV and Henipaviruses, and their related sequence, structure, and immunogenetics data**.

**bnAb**	**Viral Env target**	**PDB code**	**IGHV gene**	**IGKV/IGLV gene**	**Number of aa mutations (Identity/Similarity in percent)^a^**		**HCDR3 aa sequence (length)^b^**
					**V_H_**	**V_L_**	
b12	gp120 CD4bs	2NY7	V1-3^*^01	KV3-20^*^01	20 (80/88)	21 (78/70)	ARVGPYSWDDSPQDNYYMDV (20)
2G12	gp120 glycans	1OP5	V3-21^*^01	KV1-5^*^03	31 (68/85)	15 (83/91)	ARKGSDRLSDNDPFDA (16)
X5	gp120 CD4i	2B4C	V1-69^*^01	KV3-20^*^01	18 (83/94)	8 (92/95)	ARDFGPDWEDGDSYDGSGRGFFDF (24)
VRC01	gp120 CD4bs	3NGB	V1-2^*^02	KV3-11^*^01	41 (58/74)	28 (68/75)	TRGKNCDYNWDFEH (14)
PG9	gp120 V1/V2	3U2S	V3-33^*^05	LV2-14^*^01	19 (81/87)	15 (85/90)	VREAGGPDYRNGYNYYDFYDGYYNYHYMDV (30)
CH01	gp120 V1/V2	3TCL	V3-20^*^01	KV3-20^*^01	28 (71/86)	16 (83/90)	ARGTDYTIDDAGIHYQGSGTFWYFDL (26)
PGT128	gp120 glycans	3TV3	V4-39^*^07	LV2-8^*^01	29 (65/81)	18 (77/87)	ARFGGEVLRYTDWPKPAWVDL (21)
2F5	gp41 MPER	1TJG	V2-5^*^10	KV1-13^*^02	14 (85/91)	14 (85/96)	AHRRGPTTLFGVPIARGPVNAMDV (24)
4E10	gp41 MPER	1TZG	V1-69^*^10	KV3-20^*^01	17 (83/95)	12 (88/93)	AREGTTGWGWLGKPIGAFAH (20)
m66	gp41 MPER	ND	V5-51^*^01	KV1-39^*^01	10 (90/96)	10 (90/94)	ARQNHYGSGSYFYRTAYYYAMDV (23)
m102	Henipa sG	ND	V1-69^*^10	KV3-20^*^01	6 (94/99)	9 (91/96)	ARGWGREQLAPHPSQYYYYYYGMDV (25)
m396	SARS RBD	2DD8	V1-69^*^05	LV3-21^*^03	5 (95/95)	2 (98/99)	ARDTVMGGMDV (11)

a Number of heavy chain (V_H_) aa mutations were determined by IMGT/V-QUEST and confined to the V region only (excluding HCDR3 and Framework 4); Identity and similarity between aa sequences of bnAb and its germline counterpart were based on pairwise alignment using the Needleman-Wunsch algorithm.

b HCDR3, heavy chain complementarity determining region 3, lengths follow the CDR-IMGT definition. bnAb, broadly neutralizing antibody; CD4bs, CD4 binding site; CD4i, CD4-induced; V1/V2, variable loops V1 and V2; MPER, membrane proximal epitope region; sG, soluble G glycoprotein; RBD, receptor binding domain; PDB, Protein Data Bank; ND, not determined; IGHV, IGKV and IGLV genes are V-REGIONS from V_H_, V-KAPPA and V-LAMBDA domains respectively; aa, amino acids.

### HCDR3 length distributions, somatic V_H_ mutations and unique VDJ frequencies

**Figure 2:**
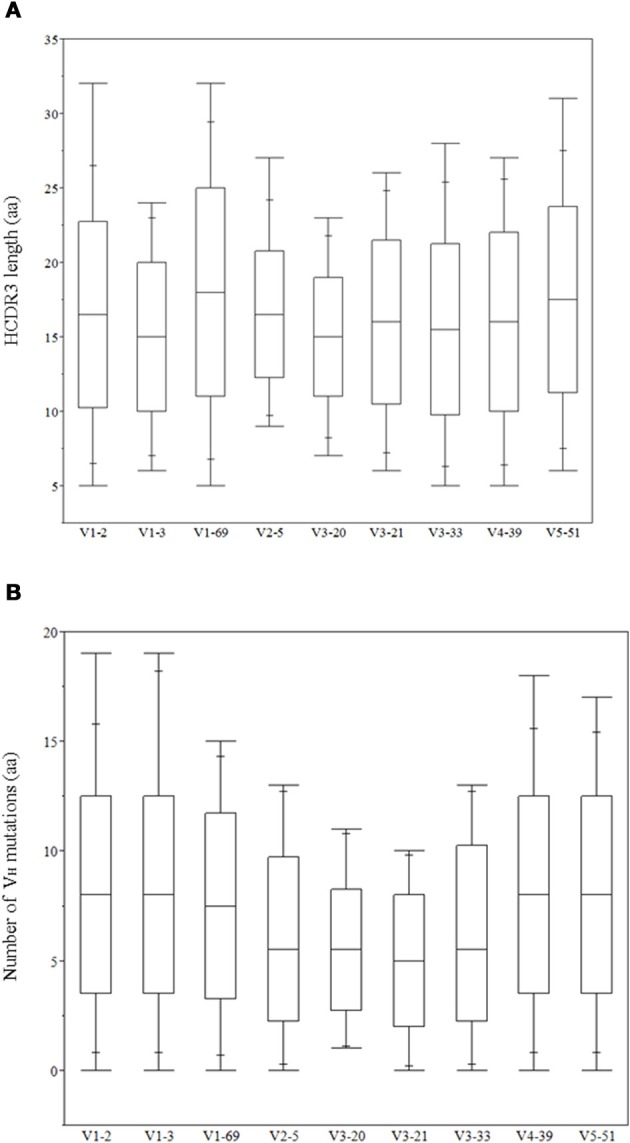
**Distribution of heavy chain complementarity determining region (HCDR3) lengths in (aa) (A) and V_H_ mutations (aa) (B) of IGHV genes related to the antiviral bnAbs**.

The role of heavy chains of antiviral antibodies in antigen recognition is found to be associated with longer HCDR3s and extensive V_H_ mutations (Table 1). Most of the bnAbs have longer HCDR3s with aa lengths ranging from 20 to 30, except for 2G12, VRC01 and m396. All of the V_H_ genes of anti-HIV-1 antibodies have a high degree of somatic mutations when compared to non-HIV-1 antiviral bnAbs. We analyzed HCDR3 length distributions and V_H_ mutations preexisting in germline-lineaged precursor antiviral antibodies from the IGHV genes of IgM repertoires from which bnAbs were generated. The box plots display the distributions of HCDR3 lengths and V_H_ mutations, Figures 2A,B, respectively, which indicates a high level HCDR3 length diversity and lesser extent of somatic mutations compared to bnAbs (Table 1).

**Figure 3:**
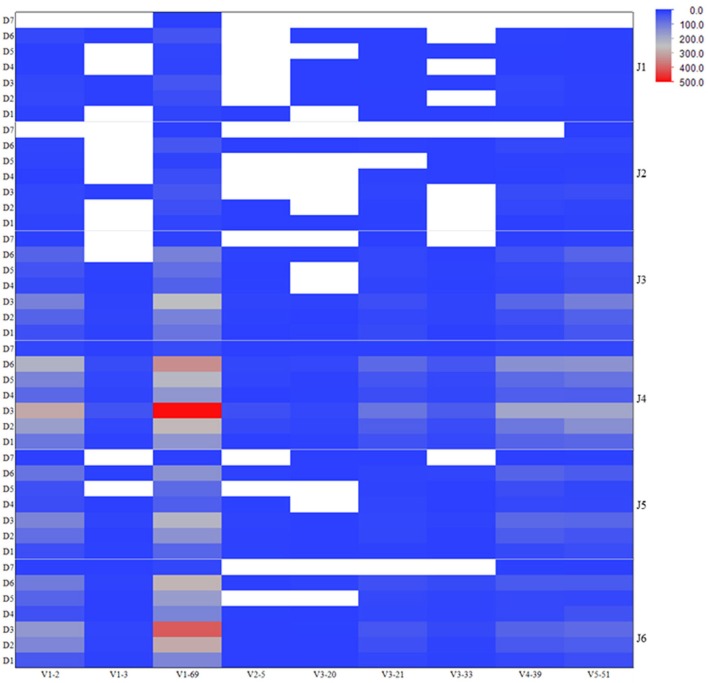
**Frequencies of VDJ recombination types as observed in the human IgM repertoire involving IGHV genes related to the antiviral bnAbs.** The heatmap is colored according to the total number of unique VDJ patterns existing in the corresponding IGHV genes used in association with different IGHD and IGHJ genes, and is shown on a blue-to-gray-to-red scale. The white-colored space represents the missed or absent VDJ recombination types in the repertoire.

To assess the VDJ repertoire usage among different antiviral related IGHV genes, we computed the frequencies of VDJ recombination patterns as observed in the V_H_ genes expressed in human IgM repertoire involving those IGHV genes of antiviral antibodies. The heatmap is shown in the Figure 3 depicting the most (red) and least (blue) abundant VDJ types existing in the germline-lineaged repertoire for the corresponding IGHV genes used in association with different IGHD and IGHJ genes. The IGHV genes V1-69 and V1-2 were frequently found to recombine with IGHJ genes J4 and J6, and IGHD genes D3 and D6.

### Identification of intermediate antiviral bnAbs and germline-linage analysis

**Figure 4:**
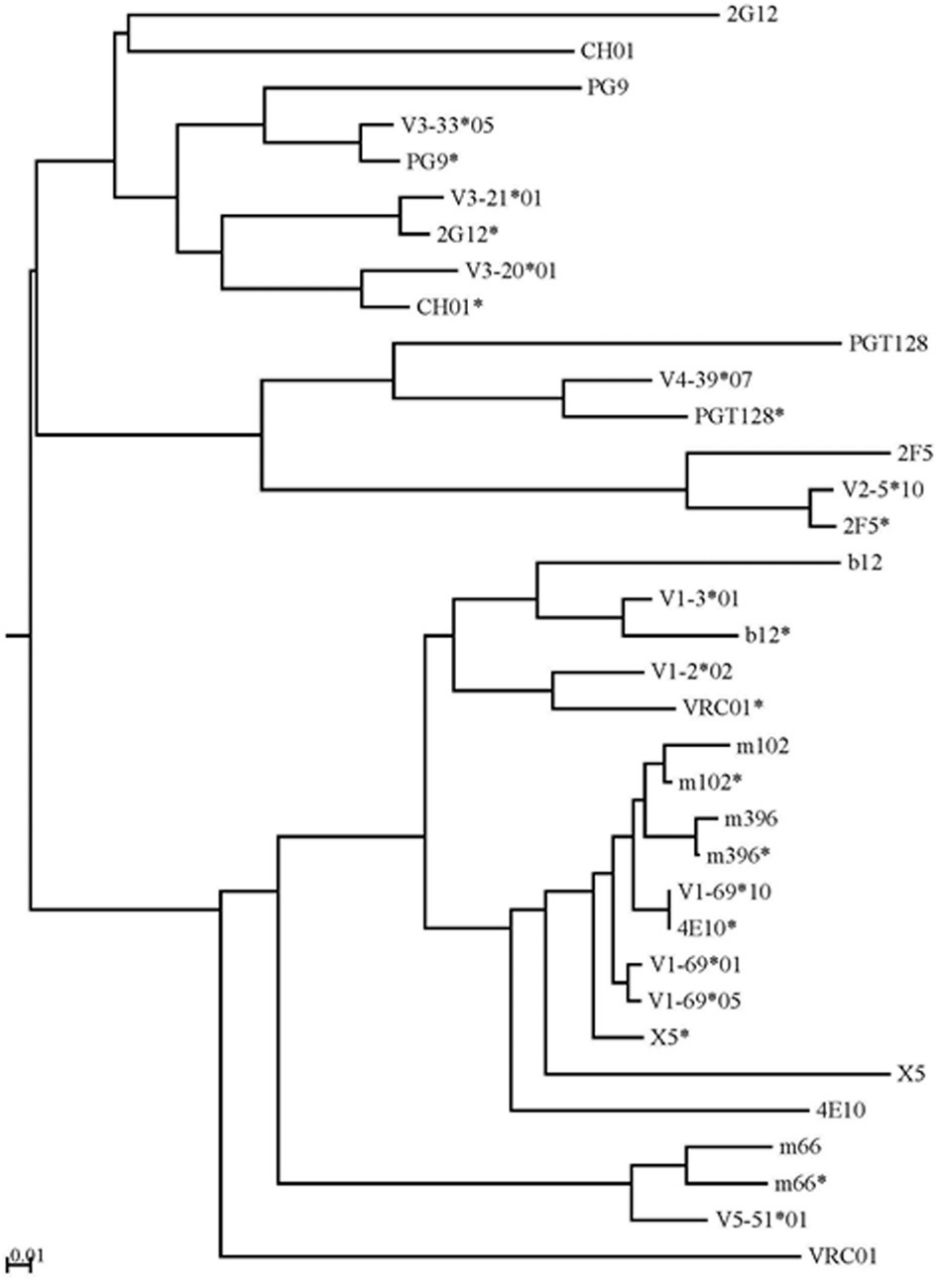
**The mid-point phylogenetic neighbor-joining tree shows the evolutionary relationships between different IGHVs of (bnAbs) with their corresponding germlines and intermediates.** The IGHV germline gene alleles follow the IMGT nomenclature and the closest intermediates of bnAbs as found from the human IgM repertoire were designated with asterisks along with names of bnAbs. Some of the anti-HIV-1 antibodies (2G12, CH01, and VRC01) were found at distal nodes in the phylogenetic tree indicating high divergence from their corresponding germline and intermediate counterparts.

The intermediate antibodies corresponding to bnAbs against the HIV-1, SARS CoV, and henipaviruses were found by analyzing the human IgM repertoire, and such intermediates with the closest similarities to the matured antiviral bnAbs were selected for germline-linage analysis by using phylogenetic method. IGHV germline gene alleles of bnAbs were obtained from the IMGT database. The mid-point phylogenetic neighbor-joining tree showing the evolutionary relationships of different antiviral antibodies with their corresponding germlines and intermediates is given in Figure 4. We observed that some of the anti-HIV-1 antibodies (2G12, CH01, and VRC01) were found at distal nodes in the phylogenetic tree indicating high divergence from their corresponding germline and intermediate counterparts. In contrast, bnAbs against SARS CoV, and henipaviruses, m396 and m102, were found closer to their intermediates.

### Analysis of intermediates of anti-HIV-1 bnAb b12 and mapping of somatic V_H_ mutations to the complex structure

**Figure 5:**
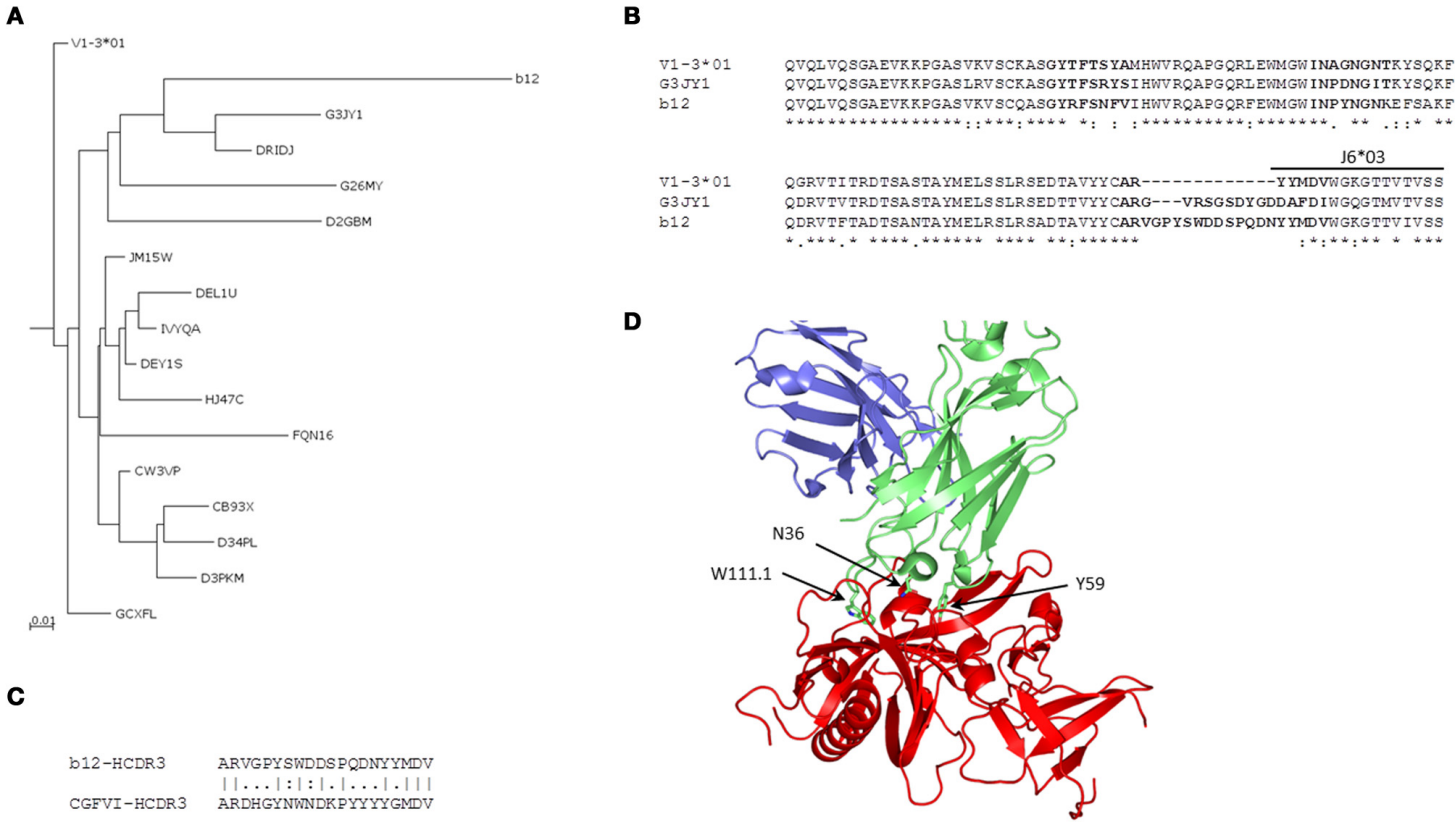
**Phylogenetic and somatic mutation analyses of anti-HIV bnAb b12. (A)** A phylogenetic sub-tree constructed from a total of 169 unique sequences from the HV1-3 subgroup, which were obtained from the 454 sequence analysis of a human IgM library, along with the heavy chain V1-3^*^01 germline and V_H_ sequence of b12 antibody showing the evolutionary relationship among different intermediates. Five-letter alphanumeric codes denote sequence labels as used in the pyrosequencing. **(B)** Multiple sequence alignment shows the differences in aa sequence between germline, intermediate, G3JY1, and b12 neutralizing antibodies. The locations of CDRs according to IMGT numbering are shown in bold face. **(C)** The most similar HCDR3 sequence to that of bnAb b12 from the IgM repertoire was found to originate from V4-b^*^01 gene and is shown in the pairwise alignment. **(D)** Mapping of three of the somatically mutated residues N36, Y59, and W111.1, as per IMGT numbering scheme, from each of the HCDRs are shown as sticks using the complex crystal structure of HIV-1 gp120-b12 (PDB code 2NY7).

We found 169 unique IGHV sequences from the V1-3 gene family as intermediates of bnAb b12 by using the 454 sequence analysis of a human IgM library. Phylogenetic analysis of those intermediates revealed two major groups, one group consisting of germline related antibodies and the other having potential intermediates closer to the bnAb b12. We then constructed a phylogenetic sub-tree selecting only the potential intermediates and the V1-3^*^01 germline along with bnAb 12. The tree was rooted at the known germline V1-3^*^01 of bnAb b12, and phylogram showed evolutionary relationship among the different intermediates (Figure 5A). One of the intermediates, G3JY1, had the maximum of 72% sequence identity (82% sequence similarity) at aa level to the bnAb b12 (Figure 5B). However, the HCDR3 length of that intermediate was found to be 17 aa long, which is 3 aa shorter than that of b12 antibody. To find the closest HCDR3 to that of b12, we scanned 28,925 unique HCDR3 sequences from the entire IgM 454 sequence data. We identified a HCDR3 with the same length (20 aa) and 50% sequence identity to that of b12 (Figure 5C), which was found to be the most similar to the HCDR3 of b12 but the IGHV gene associated with that HCDR3 was found to be V4-b. We used the HIV-1 gp120-b12 complex structure and mapped the V_H_ somatic mutations, which showed the overlapping of three mutated residues of b12 (N36 from HCDR1, Y59 from HCDR2, and W111.1 from HCDR3) that contribute to the most of binding interactions with the gp120 as previously observed (Zhou et al., 2007) (Figure 5D).

## Discussion

In this study, we have described the 454 sequence analysis of a large naïve library of human IgM antibodies, and carried out immunogenetic analysis to study the origin, diversity, and maturation of selected known bnAbs against the HIV-1, SARS CoV RBD, and henipaviruses sG proteins. We have found intermediates of antiviral related bnAbs, of which most of those against the HIV-1 were highly diverged from their mature forms of bnAbs as compared to other viral targets, SARS CoV, and henipaviruses.

Although antibodies are generated through various mechanisms involving VDJ recombination, junctional modification, and hypermutations, the V-genes sculpt the most of the antigen-combining sites, CDR1 and CDR2, and support frameworks for the CDR3. We found that antiviral antibodies targeting different Env binding regions of the HIV-1 and other viruses utilized different germline V-genes as the origins (Table 1). We noted that, among antiviral-related bnAbs, the V1-69 gene usage was dominated in the heavy chains while V3-20 and V2-14 genes of kappa and lambda were used with the highest frequencies in the light chains of human IgM repertoire (Figure 1). Accordingly, four of the V_H_ genes of bnAbs (4E10, X5, m102, and m396) originated from the V1-69, and three of them paired with the kappa V3-20 gene. One possible reason for dominance in the usage of those germline genes could be reflecting from the relatively higher frequencies of distributions observed in the expressed IgM repertoire (Figures 1A,B). The HV3 gene was used in the three of the HIV-1 bnAbs, 2G12, PG9, and CH01. The structural data for most of the bnAbs selected in this analysis were known and the heavy chains of these bnAbs were dominantly used. The increased number of V_H_ mutations and longer HCDR3s are characteristics for the HVI-1 bnAbs when compared to other antiviral bnAbs (Breden et al., 2011). We analyzed the distribution of HCDR3 lengths and extent of somatic V_H_ mutations in the human IgM repertoire to compare with that of antiviral-related bnAbs (Figure 2). The results showed that the longer HCDR3s and low level of somatic V_H_ mutations as compared to the HIV-1 bnAbs existed in the intermediates as found from the 454 sequencing. The somatic diversity through VDJ recombination involving antiviral-related V-genes in the IgM repertoire was found high; the most abundant VDJ combination consisted of the HV1-69 gene with certain D and J genes as depicted in gray and red (Figure 3), which might be the reason for the preferential usage of that HV1-69 in many other viral diseases (Sui et al., 2009).

Further, bnAbs against the SARS CoV and henipaviruses shared the heavy chain V-gene germline, HV1-69, with two of the HIV-1 bnAbs, 4E10, and X5. All of these four bnAbs were less divergent from their V-germlines and intermediates, when compared to other HIV-1 bnAbs, and formed a single cluster at a mid-point rooted phylogenetic tree (Figure 4). The gp41 membrane-proximal epitope region (MPER) binding site bnAbs, 2F5, and m66, were moderately divergent from their V-germlines and intermediates and formed distinct clusters. The V-gene of VRC01 bnAb was the most divergent from its respective germline as well as the closest intermediate, and was placed at a distal branch of HV1 subgroup of bnAbs. For the mid-point rooted phylogenetic analysis, we included the closest intermediates only; however, favored maturation pathways could involve other intermediates too. We created the germline-rooted phylogenetic tree as a use-case for the bnAb b12 (Figure 5A) and analyzed the maturation pathway along different V-gene intermediates from HV1-3 gene family. The closest b12 intermediate, designated as G3JY1, had three mutations each at HCDR1 and HCDR2 compared to the germline, and were found similar though not identical to that of mature b12 (Figure 5B). Interestingly, we also identified a HCDR3 with the same length (20 aa) and 50% sequence identity to that of b12 (Figure 5C), which was found to be the most similar to the HCDR3 of b12 but the IGHV gene associated with that HCDR3 was found to be V4-b. This might suggest for the possible maturation mechanism of bnAbs which could be involving the VH replacement (Chen et al., 1995). These two mutated residues (N36 from HCDR1 and Y59 from HCDR2) from the V-gene and a Trp residue from the D-gene (W111.1 from HCDR3) contributed to the most of binding interactions with the gp120 (Figure 5D) (Zhou et al., 2007).

In summary, the 454 sequence analysis of a large naïve human antibody repertoire corresponding to the selected antiviral-related bnAbs revealed the germline V-gene usage, VDJ rearrangement, HCDR3 length diversity, and somatic mutations of potential intermediate antibodies of HIV-1 and other viruses such as SARS CoV and henipaviruses. Thus, B cell germline-lineage analysis using the 454 sequence data from different sources could help finding appropriate antibody intermediates, pathways, and mechanisms useful in the development of bnAbs and vaccines against the HIV-1 and other viral diseases.

### Conflict of interest statement

The authors declare that the research was conducted in the absence of any commercial or financial relationships that could be construed as a potential conflict of interest.
